# p27^Kip1 ^deficiency promotes prostate carcinogenesis but does not affect the efficacy of retinoids in suppressing the neoplastic process

**DOI:** 10.1186/1471-2407-10-541

**Published:** 2010-10-08

**Authors:** Winna Taylor, Amanda Mathias, Arshia Ali, Hengning Ke, Nikolay Stoynev, Anne Shilkaitis, Albert Green, Hiroaki Kiyokawa, Konstantin Christov

**Affiliations:** 1 University of Illinois at Chicago, Department of Surgery, Division of Surgical Oncology, 840 S. Wood St, Chicago, IL 60612 USA; 2 Laboratory of Molecular Toxicology, National Institute of Environmental Health Sciences, Research Triangle Park, NC 27709, USA; 3 Department of Endocrinology, Medical University, ul. Zdrave No.2, Sofia, Bulgaria; 4 Department of Molecular Pharmacology and Biological Chemistry, Robert H. Lurie Comprehensive Cancer Center, Feinberg School of Medicine, Northwestern University, 303 E. Superior, Lurie 3-113 Chicago, IL 60611 USA

## Abstract

**Background:**

p27 is a cell cycle suppressor gene, whose protein is a negative regulator of cyclin/cdk complexes. p27 is also a potential target of retinoids in cancer prevention studies. In benign prostate hyperplasia (BPH), and in most carcinomas, p27^Kip1 ^is down-regulated, suggesting its potential resistance to retinoids. To test this hypothesis, we examined the efficacy of 9-cis retinoic acid (9cRA) to suppress prostate cell proliferation (PECP) and carcinogenesis in p27^Kip1 ^deficient mice.

**Methods:**

p27^Kip1 ^deficient (-/-), heterozygous (+/-) and homozygous (+/+) mice were treated for 7 days with testosterone, 9cRA, or with both, and cell proliferation in dorsolateral prostate (DLP) was determined by BrdU labeling. Prostate carcinogenesis was induced by N-Methyl-N-Nitrosourea (MNU) and hormone stimulation.

**Results:**

PECP in DLP of two-month-old mice of all genotypes was similar but significantly increased in old p27-/- mice only. Testosterone treatment increased PECP in all three p27 genotypes with the highest values in p27-/- mice. p27^Kip1 ^deficiency did not affect the response of PEC to 9cRA and to 9cRA+testosterone. The decrease of p27^Kip1 ^in p27+/- and p27-/- mice progressively increased the incidence and frequency of PIN and tumors. 9cRA suppressed PIN in all three p27 genotypes and this was associated with decreased PECP and increased cellular senescence.

**Conclusions:**

This data indicates that p27^Kip1 ^deficiency promotes prostate cell proliferation and carcinogenesis but does not affect 9cRA's potential to suppress prostate carcinogenesis, suggesting that patients with PIN and carcinomas lacking or having a low level of p27^Kip1 ^expression may also benefit from clinical trials with retinoids.

## Background

Various risk factors, such as race (with black men having the highest risk), family history, and genetic predisposition appear to play principal roles in the development and progression of prostate cancer [1-3]. Over the last several years, increasing attention has been paid to the role of p27^Kip1 ^expression in the development and progression of various tumors, including prostate cancer. Human tumors lacking p27^Kip1 ^appear more malignant than those with high levels of the gene's expression [4,5]. Normal prostate epithelial cells (PEC) exhibit abundant amounts of p27^Kip1 ^protein and mRNA, whereas in benign prostate hyperplasia (BPH) p27^Kip1 ^decreases to undetectable levels. In contrast to BPH, most prostate carcinomas contain p27 mRNA but low to undetectable levels of p27^Kip1 ^protein, suggesting post-transcriptional alterations in the gene's activity [6,7]. Little is known regarding when in the course of prostate carcinogenesis disassociation between p27 mRNA and protein expression occurs or whether p27^Kip1 ^alone or in cooperation with other genes is involved in mediating the response of prostate pre-malignant and tumor cells to various chemopreventive and antitumor agents.

p27^kip1 ^is a cell cycle suppressor gene, whose protein product is a negative regulator of cyclin dependent kinases (CDKs) [8-10]. Cyclin dependent kinases-2/4/6 (CDKs) selectively bind to cyclin D1- D3, E, A, B, forming complexes that are variably expressed during the cell cycle. When inhibited by p27^Kip1^, p21^Wef1/Cip1^, or p16^Ink4a^, CDKs can suppress cell cycle progression by modulating pRb phosphorylation, leading to inhibition of E2F transcription factors and further to suppression of DNA replication [11,12]. p27^Kip1 ^may also cooperate with other cell cycle suppressor genes and thus further inhibit cell proliferation and carcinogenesis [13,14]. For example, 100% of mice deficient in both p27^Kip1 ^and PTEN (phosphatase and tensin homolog deleted from chromosome 10) (PTEN +/-; p27-/-) developed prostate tumors within 4-6 months vs. 50% of those with the PTEN mutation only [15-17]. Recently, Besson et al. [18] discovered an oncogenic activity of p27^Kip1 ^that causes stem cell expansion and a multiple tumor phenotype. They generated a knock-in mouse in which four amino acid substitutions in the CDKN1b gene product prevented its interaction with cyclins and CDKs (p27^CK^*) and found tumors in multiple organs, including: lung, pituitary, retina, adrenals, ovary, spleen, and lymphomas. No data has been published on the effects of p27^Kip1 ^deficiency on chemically-induced prostate carcinogenesis and on the sensitivity of PEC to retinoids.

Studies from the Fero et al. [19] group have shown that p27-/- and, to a lesser extent, p27+/- mice are more susceptible than p27+/+ mice to radiation and chemically induced carcinogenesis. They have found that partial reduction in p27 expression in p27+/- mice can also sensitize cells in a tissue specific manner to undergo malignant transformation. However, they did not examine prostate glands (personal communication). In human tumors haplo-insufficiency is not a frequent phenomenon. However, a moderate decrease in protein expression of certain tumor suppressors, including p27^Kip1^, may also promote the neoplastic process [20].

Most chemopreventive and antitumor agents, including retinoids, affect normal and tumor cells by inhibiting cell proliferation, and this has been associated with increased expression of cell cycle suppressors [21]. It has been suggested that retinoids which induce cell differentiation and suppress cell proliferation can up-regulate p27^kip1 ^and thereby inhibit cell cycle progression [22]. Others [23] and we [24] recently observed that 9cRA can suppress prostate carcinogenesis when given to rats treated with a carcinogen or to transgenic mice that spontaneously develop prostate tumors [25]. Our previous studies on Noble rats revealed that, among various retinoids, 9cRA but not 4-(Hydroxyphenyl) retinamide (4-HPR) reduced the incidence and multiplicity of prostate intraepithelial neoplasia (PIN) [24]. The molecular mechanisms involved in inhibition of prostate carcinogenesis by 9cRA and other retinoids are poorly understood. 9cRA is a ligand of both retinoic acid receptors (RARs α,β,γ) and retinoid × receptors (RXRs α,β,γ), suggesting receptor dependent mechanisms of cell and tumor growth inhibition [22]. However, retinoids may also affect normal and tumor cells by receptor independent pathways [26,27].

In this study we assessed the role of p27 deficiency on prostate cell proliferation, response to hormonal stimulation, and to treatment with retinoids. We also examined whether p27^Kip1 ^deficiency may affect MNU-induced prostate carcinogenesis and the efficacy of 9cRA in suppressing the neoplastic process. We found that p27^Kip1 ^deficiency promotes prostate cell proliferation and carcinogenesis but does not affect the efficacy of 9cRA in suppressing PIN and tumor development.

## Methods

### Mice

p27+/+, p27+/- and p27-/- mice were generated in our lab by crossbreeding p27+/- female with p27-/- male mice. p27 deficient mice (+/- and -/-) were obtained from Kiyokawas' lab and have been developed based on the c57BL/6 mouse background. Mouse genotype was assessed by PCR using DNA isolated from small tail tissue samples when the animals were 14 days old, as described previously [28]. For the long-term carcinogenesis study, male p27+/+, p27+/- and p27-/- mice were followed for up to 12 months. Most p27-/- mice died from pituitary and intestinal tumors at the age of 7 to 12 months, as has been reported previously [19,28]. The animals were housed at the Biology Research Laboratory at the University of Illinois, Chicago, and their breeding and treatment were conducted according to the requirements listed in the Guide for the Care and Use of Laboratory Animals issued by the University's Animal Care Committee. Animals had free access to water and food, Teklad 4% Purina mouse/rat diet (Harland Teklad, Madison, WI).

### Hormone stimulation of prostate cell proliferation

Two separate studies were performed. In the short-term study, two-month-old mice of all three p27 genotypes were treated for 7 days with testosterone (Sigma Chem. Co., St. Louis) mixed in purified sesame oil. Each animal received 3 sc injections of testosterone, 1.0 mg/kg body weight, 2 days apart in a volume of 0.1 ml. In the long-term chemoprevention study, two experiments were performed. In the first, two-month-old p27+/+, p27+/- and p27-/- mice were first treated with testosterone, as described above, and then, on the sixth day after initiation of hormone stimulation, MNU was injected ip. In the second chemoprevention experiment, mice were first sc implanted with a testosterone pellet (12.5 mg/pellet: Cat No: SA-151, Innovative Research of America, Saratoga, FL). Six days later the animals were ip injected with MNU to initiate the neoplastic process. Four days after carcinogen administration the animals received sc an estradiol pellet (0.18 mg/pellet: Cat No: SE-121, Innovative Research of America, Saratoga, FL), aiming to further stimulate PEC proliferation, as was reported previously [23,24]. However, in the second experiment, within four weeks, about 50% of p27+/- and p27-/- animals developed severe prostate hypertrophy, leading to almost complete obliteration of urethra, causing urine retention in the bladder and death by kidney failure.

### Carcinogen

N-Methyl-N-Nitrosourea (MNU), was obtained from Ash Stevens Inc. (Detroit, MI), dissolved in sterile acidified saline (pH 5.0), and injected ip at 30 mg/kg body weight six days after sc testosterone treatment or implantation of testosterone pellets. At that time, the number of proliferating PEC sharply increased (see Table 1), suggesting that a high number of proliferating cells would facilitate MNU-induced prostate carcinogenesis.

**Table 1:** Effects of testosterone and 9cRA on cell proliferation in DLP

	Controls		9cRA		Testosterone		Testosterone+9cRA	
Genotype	No	BrdU (%)	No	BrdU (%)	No	BrdU (%)	No	BrdU (%)
p27+/+	5	0.3 ± 0.2	6	0.3 ± 0.2	5	0.7 ± 0.3 **	6	0.7 ± 0.3 **
p27+/-	6	0.3 ± 0.2	5	0.5 ± 0.4	8	1.5 ± 0.6* **	6	1.4 ± 0.5* **
p27-/-	6	0.4 ± 0.2	6	0.5 ± 0.3	9	1.7 ± 0.3* **	5	1.5 ± 0.3* **

Two-month-old p27+/+, p27+/-, and p27-/- mice were treated for 7 days with: placebo (sesame oil-control group); 9cRA, 30 mg/kg body weight; testosterone, 1.0 mg/kg body weight; or combination of 9cRA + testosterone. BrdU, at 50 mg/kg body weight was injected 2 hrs prior to animal's sacrifice. BrdU-labeled cells were evaluated among epithelial cells of DLP by counting at least 500 cells per animal. Two to three levels of sectioning, 30 microns apart, were employed in some animals to reach the above number of cells. The evaluation of BrdU-labeled cells was done blindly, without knowledge of the animals' genotype and the treatment protocol. No, number of animals in each group; *, significant difference (p < 0.05) as compared to the values in p27+/+ mice; **, significant difference (p < 0.05) as compared to control values.

**9-cis retinoic acid (9cRA) **was obtained from the repository of the National Cancer Institute. It was suspended in purified sesame oil (vehicle) and given by gavage by #20 gauge needle in 0.1 ml 6 days a week at a concentration of 30 mg/kg body weight. For the long-term chemoprevention study, 9cRA was mixed in the diet at 50 mg/kg.

### Histomorphology

Mouse prostate consists of 3 glands: dorso-lateral (DLP), anterior (AP, coagulating gland) and ventral prostate (VP). Seminal vesicles were also macroscopically examined as part of the prostate tissue complex. During autopsy all the above glands were removed, separated under a dissection microscope from the urinary bladder and urethra, fixed in 10% formalin, and embedded in paraffin [29]. DLP and AP were cut longitudinally through the urethral part of the prostate and both tissue pieces were put in the cassette with the cutting area facing down. Stepwise, 4 μm thick paraffin sections at three separate tissue levels 30 μm apart were cut. On each slide 3 tissue sections were mounted. All resulting slides were labeled consecutively. This method of embedding and sectioning has proved to be essential for detecting small lesions and micro invasive carcinomas and accurately tracing their origin and structural characteristics.

### RARα and RXRα expression

Both receptors were determined by immunocytochemistry employing, respectively, anti-RARα (SC-551, Santa Cruz Biotech.) and anti-RXRα (SC-553) antibodies and ABC kit. Only cells with staining localized in the nucleus were considered positively stained.

### Proliferating cells

In the short-term study, proliferating cells were identified by BrdU-labeling. Two hrs prior to sacrifice, mice were ip injected with 5-Bromodeoxyuridine (BrdU), 50 mg/kg body. BrdU-labeled cells were detected by their corresponding antibody (Santa Cruz Biotech. Inc., CA) and ABC kit, as we reported previously [28]. In the long-term cancer prevention study, proliferating cells were identified at the end of experiment by Ki-67 antibody (Santa Cruz Biotech. Inc.) and ABC-kit.

### Apoptosis

Apoptotic cells were identified on parallel tissue sections by TUNEL assay, as recommended by the ApopTag *in situ *hybridization detection kit (Oncor, Co., Gaithersburg, MD). Tissue sections were counterstained by methyl green for visualization of tumor morphology.

### Senescent cells

An SA-β-Gal activity assay was employed to identify senescent cells in prostate tissues and tumors [30]. Frozen sections (5-7 μm thick) from control and cells treated with 9cRA were fixed in 3.0% formaldehyde for 5 min, washed in PBS, and stained in β-Gal (5-bromo-4-chloro-3-indolyl-β-D-galactosidase) (Sigma Chem., Co. St. Louis, MO) solution at pH 6.0 for 24 hrs at 37°C.

### Statistical analysis

Differences in the incidence of prostate lesions among various groups were evaluated by the ANOVA test followed by the LSD test. Differences in the average numbers of tumors were determined by ANOVA followed by unpaired Student's T-test. SAS software was used for statistical analysis. Differences with p < 0.05 were considered significant.

## Results

### p27^Kip1 ^deficiency increases prostate epithelial cell proliferation

To determine whether the decrease of p27^Kip1 ^in both p27 heterozygous and p27 deficient mice may affect PEC proliferation, two-month-old p27+/+, p27+/- and p27-/- mice were injected ip with BrdU two hrs prior to sacrifice and proliferating cells identified by a corresponding anti-BrdU antibody. DLP was predominantly examined, because most PIN and carcinomas in human prostate develop in tissue areas relevant to DLP of mice. As shown in Table 1, the percentage of BrdU-labeled cells in DLP of mice with the above p27 genotypes is very low (< 0.5%), lacking any significant difference in the values between them. 9cRA at 30 mg/kg body weight given for 7 days by gavage did not significantly affect proliferation of normal PEC. Since testosterone plays a significant role in the development and progression of human prostate cancer, we determined whether p27^Kip1 ^deficiency may affect testosterone-induced cell proliferation. Two-month-old p27+/+, p27+/- and p27-/- mice were treated for 7 days with placebo, testosterone, or a combination of testosterone + 9cRA, and BrdU-labeled cells were determined (Table 1). In testosterone-treated animals, BrdU-labeled cells increased in all three p27 genotypes; the most significant increases were in p27+/- and p27-/- mice, from 0.3 ± 0.2% (placebo) to 1.5 ± 0.6% (p < 0.02) in the former and from 0.4 ± 0.2% (placebo) to 1.7 ± 0.3% (p < 0.001) in the latter. The values of BrdU-labeled cells in testosterone-treated animals were significantly higher in p27+/- and p27-/- mice than in p27+/+ mice. This data indicates that either p27 haplo-insufficiency or p27 deficiency can promote testosterone-induced cell proliferation. In animals treated with testosterone + 9cRA, the values of BrdU-labeled cells were close to those of animals treated with testosterone only and there was no significant difference between them for all three p27 genotypes. However, proliferating cells in p27+/- and p27-/- treated with testosterone + 9cRA were higher than those in p27+/+ mice (Table 1), further indicating that p27 deficiency can promote proliferation of prostate epithelial cells.

### p27^Kip1 ^deficiency promotes prostate carcinogenesis

**Figure 1:**
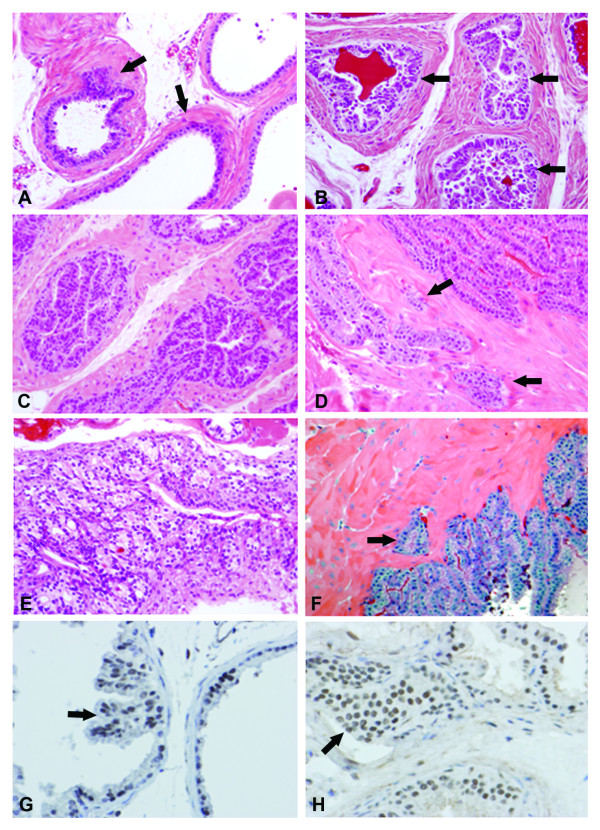
**Morphological changes in DLP of p27 deficient mice in the course of carcinogenesis**. 1A: Dorso-lateral prostate of 6-month-old p27-/- mice. Note the glandular structures are covered by a single layer of prostate epithelial cells (PEC). Glands are surrounded by a thick layer of myoepithelial cells, stroma cells and collagen (arrows). H&E staining × 200. 1B: High grade PIN in a p27-/- mice treated with MNU and sacrificed at the age of 9 months. Hyperplastic and dysplastic PEC cover entire glands and form papillary and alveolar structures (arrows). The surrounding connective tissue stroma is very abundant (arrow had). H&E × 200. 1C: Ductal carcinoma of papillary type in DLP of a p27-/- animal treated with MNU and sacrificed at the age of 10 months. Note, tumor cells occupy the entire glands lumen. H&E × 200. 1D: Infiltrative prostate carcinoma in a p27-/- animal sacrificed at the age of 12 months. Tumor cells infiltrate the surrounding stroma (arrows). H&E × 200. 1E: Pituitary tumor in a p27-/- mice sacrificed at the age of 10 months. H&E × 200. 1F: Intestinal carcinoma in a p27-/- mice sacrificed at the age of 9 months. Note tumor cell infiltration in the surrounding stroma (arrow). H&E × 200. 1G: RARα expression in DLP of a 6-month-old p27+/- mice. RARα was detected by anti-RARα antibody (SC-251) and ABC kit. The receptor is expressed in papillary structures (arrow) and in the basal layer of epithelial cells. The slide is counterstained by hematoxylin × 200. 1H: RXRα expression in PIN of p27-/- 9-month-old mice. Note that most epithelial cells express RXRα receptor (arrow). The slide is counterstained by hematoxylin × 200.

To assess the role of p27 deficiency on prostate carcinogenesis, p27+/+, p27+/- and p27-/- mice at the age of two months were sc injected with testosterone, as described previously, to stimulate cell proliferation, and six days latter MNU was administered ip to initiate the neoplastic process. Four days after MNU administration, the animals were randomized in control (MNU-treated) and 9cRA-treated (MNU+9cRA) groups. The latter animals were treated with 9cRA as long as they remained alive. Most p27-/- mice died at the age of 7 to 12 months primarily from pituitary and other tumors: intestinal, kidney, thymus. Therefore, the incidence and frequency of PIN and tumors in all three genotypes were examined and compared within this time frame. Male p27-/- mice at the end of the study were larger in size as compared to p27+/+ mice, whereas p27 +/- mice had intermediate body weight (Table 2). The weight of DLP also increased in p27+/- and further increased in p27-/- mice as compared to p27+/+ mice: from 48.3 ± 13.1 mg in p27 +/+ to 67.2 ± 14.5 mg in p27+/- to 97.6 ± 21.0 mg (p < 0.01) in p27-/-. MNU alone induced PIN in all three p27 genotypes (Table 3). PIN was detected the most in DLP (Fig. 1A-B), where intraductal (Fig. 1C) and invasive (Figs. 1D-arrows) carcinomas were also identified. The incidence and multiplicity of PIN progressively increased, respectively, from 11% and 0.16 ± 0.5 in p27+/+ to 60% and 1.5 ± 0.5 (p < 0.05) in p27+/-, and further to 70% and 2.0 ± 0.4 in p27-/- (p < 0.005) mice (Table 3). Multiplicity of PIN in p27-/- mice was also higher as compared to that in p27+/- mice (p < 0.01). In addition to PIN in DLP, MNU also induced tumors in distant organs (DO): (pituitary, thyroid, lymph node, intestine) of both p27+/- and p27-/- mice.

**Table 2:** Alteration in body weight and in DLP weight in the course of prostate carcinogenesis

Genotype	Body wt: (g) Placebo		Body wt. (g) 9cRA		DLP-(mg) Placebo	DLP-(mg) 9cRA
p27+/+	8	30.8 ± 4.3*	10	27.2 ± 5.4*	48.3 ± 13.1**	45.6 ± 7.7**
p27+/-	10	35.8 ± 5.1	12	31.3 ± 5.6	67.2 ± 14.5	60.2 ± 10.5
p27-/-	12	41.4 ± 5.6*	10	35.5 ± 6.4*	97.6 ± 21.0** x	80.5 ± 18.6** x

These are the final measurements of body weight (g) at the end of experiment when the animals were sacrificed. DLP was isolated at autopsy, measured on an analytical balance and then frozen or embedded in 10% neutral formalin. Body and DLP weights were higher in p27-/- as compared to p27+/+ mice; *, p < 0.01; **, 0.001. 9cRA also decreased DLP weight in p27-/- mice (x) as compared to placebo-treated mice (p < 0.02).

**Table 3:** 9cRA inhibits MNU-induced prostate carcinogenesis in p27 deficient mice

			PIN		P	Tumors	
Genotype	Animals No	9cRA	Incidence	Frequency		P	DO
p27+/+	18	0	11	0.16 ± 0.5		0	0
p27+/+	17	+	0	0	0	0	0
p27+/-	16	0	60	1.5 ± 0.5*		3	4
p27+/-	15	+	44	1.1 ± 0.6	0.05	2	4
p27-/-	15	0	70	2.0 ± 0.4*		3	6
p27-/-	15	+	53	1.4 ± 0.5	0.005	4	6

Animals were treated with MNU, 30 mg/kg body weight at the age of 2 months and followed until sacrifice between 7-12 months of age. PIN were identified in DLP by cutting longitudinally through the urethral part of the prostate. H&E staining was used for identification of PIN. Stepwise, 4 μm thick paraffin sections at three separate tissue levels 30 μm apart were cut and the number of PIN per prostate determined. Incidence, percentage of animals with PIN per group; frequency, number of PIN per animal. PIN were determined in various groups without knowledge of the treatment protocol. (P), prostate; (DO), distant organs; *, significant difference (p < 0.01) in the multiplicity of PIN between p27+/- and p27-/- mice;

### 9cRA suppresses prostate carcinogenesis irrespective of p27^Kip1 ^expression

Treatment of animals with 9cRA for 7-12 months reduced by 10-15% the body weight of all three p27 genotypes, with lack of significant difference between control, placebo-treated and 9cRA-treated animals (Table 2). 9cRA did not significantly decrease DLP weight in p27+/+ and p27+/- mice, but reduced DLP weight in p27-/- mice, from 97.6 ± 21.0 mg in placebo to 80.5 ± 18.6 mg (p < 0.02) in 9cRA-treated mice (Table 2). 9cRA reduced PIN in all three p27 genotypes. Thus, in p27+/+ mice PIN decreased from 11% for incidence and 0.16 ± 0.5 for frequency in placebo-treated to zero in 9cRA-treated animals. In these animals no prostate or distant tumors were detected (Table 3). 9cRA was also efficacious in both p27+/- and p27-/- mice. In p27+/- mice, 9cRA decreased PIN from 1.5 ± 0.4 (placebo) to 1.1 ± 0.6 (p < 0.05), and in p27-/- mice from 2.0 ± 0.4 (placebo) to 1.4 ± 0.4 (p < 0.005). 9cRA apparently was not efficacious and did not reduce the number of tumors in distant organs (Table 3).

### 9cRA was unable to suppress hormone-induced prostate carcinogenesis

We also determined whether testosterone + estradiol hormone stimulation might promote MNU-induced carcinogenesis in p27 deficient mice and affect the chemopreventive efficacy of 9cRA. In previous studies, others [23] and we [24] have shown that the combination of carcinogen + testosterone + estradiol stimulates prostate carcinogenesis in rats. For this study, a testosterone pellet was first sc implanted to simulate cell proliferation and 6 days later the animals were injected with MNU to initiate prostate carcinogenesis. Four days after carcinogen administration an estradiol pellet was sc implanted. Both testosterone and estradiol pellets were designed to release hormones within 60 days, with a second and third pellet projected to be implanted after those initial 60 days. However, testosterone + estradiol treatment induced severe prostate hypertrophy, predominantly in p27-/- and p27+/- mice, with complete blockage of urethra that caused urine retention in the bladder, and about 50% of animals died within 4-6 weeks due to kidney failure and intoxication. Therefore, the number of animals in individual groups at the end of the experiment (Table 4) is relatively small, as compared to 20 animals per group at the beginning of the experiment. The experiment continued with surviving animals, and no second or third pellets were implanted. As shown in Table 4, PIN was identified in all p27 genotypes, with the lowest value in p27+/+ (1 in 15 animals), and its incidence and frequency increased in p27+/- (40% for incidence and 0.7 ± 0.3 for frequency) and progressed further in p27-/- mice (80% for incidence and 2.3 ± 1.2 (p < 0.05) for frequency). Tumors localized in prostate and distant organs (DO) were also detected in p27+/- (No: 7) and in p27-/- (No: 10) mice. Treatment of animals with 9cRA was less efficacious than in animals not undergoing hormone stimulation. Thus, the incidence of PIN was similar in placebo- and 9cRA-treated p27+/+ and p27-/- mice, 13% in both placebo groups and 40% and 60% in treated animals, respectively (p < 0.5); even in 9cRA-treated mice a tendency for increase of PIN multiplicity was observed, rising from 0.7 ± 0.3 in placebo to 1.5 ± 1.1 (p < 0.1). In p27-/- mice hormone stimulation for 60 days further increased PIN multiplicity (2.3 ± 1.2 per animal) as compared to p27+/+ and p27+/- mice, but 9cRA was again not efficacious in suppressing the neoplastic process (Table 4).

**Table 4:** Incidence and frequency of PIN and tumors in mice treated with MNU + testosterone + estradiol

Genotype	9cRA	Animals-No		PIN		P	Tumors* No
		at start	at end	Incidence	Frequency		
p27+/+	0	20	15	13	0.13 ± 0.3		2
p27+/+	+	20	15	13	0.13 ± 0.3	ns	1
p27+/-	0	20	10	40	0.7 ± 0.3		4
p27+/-	+	20	10	60	1.3 ± 1.1	ns	3
p27-/-	0	20	10	80	2.3 ± 1.2		5
p27-/-	+	20	8	87	1.7 ± 1.0	ns	5

Animals were implanted with a testosterone pellet at the age of 2 months and 6 days later MNU was injected ip to initiate the neoplastic process. Four days after carcinogen administration an estradiol pellet was sc implanted. %, incidence of tumors among control and treated animals; No: multiplicity of PIN per treated animal; ns, not significant (p > 0.05). To determine the incidence (%) and frequency of PIN in DLP, 4 μm thick longitudinally paraffin sections through the urethral part of the prostate were cut stepwise at three separate levels 30 μm apart.

### 9cRA inhibits prostate cell proliferation and induces cellular senescence

**Figure 2:**
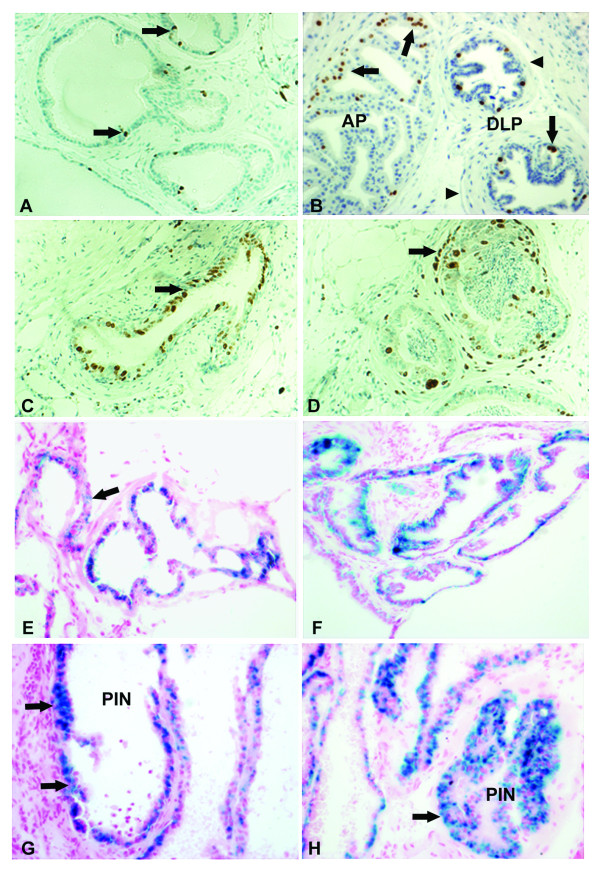
**Effects of 9cRA on cell proliferation and senescence in DLP of p27 deficient mice**. Fig. 2A: Cell proliferation, as determined by Ki-67 antibody in DLP of 9-month-old p27+/+ mice. Note several Ki-67-positive cells (brawn-stained, arrows) in glandular structures which are covered by a single layer of epithelial cells. The slide is counterstained by hematoxylin × 200. 2B: Ki-67 positive cells (arrows) in anterior (large left) and DLP (two right glands) of 9-month-old p27-/- mice. Note the thick connective tissue surrounding glandular structures. The slide is counterstained by hematoxylin × 200. 2C: Ki-67 positive cells in PIN of MNU-treated mice. The number of Ki-67 positive cells is higher (arrow), as compared to those in Fig. 2B. The slide is counterstained by hematoxylin × 200. 2D: Treatment of p27-/- mice with 9cRA for 6 months reduced the number of Ki-67 positive cells in PIN (arrow). Note the inflammation associated cells in PINs' lumen. The slide is counterstained by hematoxylin × 200. 2E: Senescent cells (blue stained) determined by SA-β-Gal staining in glandular and ductal structures of DLP of 9-month-old p27+/- mice. Senescent cells are also detectable among myoepithelial and stroma cells (arrows). The slide is counterstained by nuclear fast red × 200. 2F: Treatment of animals with 9cRA increased senescent cells (blue stained) in DLP and AP of a p27+/- mice. The slide is counterstained by nuclear fast red × 200. 2G: 9cRA increased senescent cells in low grade PIN (arrows) as compared to non-PIN areas of a 10-month-old p27+/- animal. The slide is counterstained by nuclear fast red × 200. 2H: 9cRA also increased senescent cells in high grade PIN as shown in the right glandular structure. The slide is counterstained by nuclear fast red × 200.

Since 9cRA can modulate cell growth by retinoid receptor dependent and independent mechanisms, RARα and RXRα were also determined by ICH. These two receptors are chief targets of 9cRA in epithelial cells [22]. As shown in Fig 1G, RARα is expressed in prostate epithelial cells of non-PIN (right gland), as well as in the papillary structures of PIN (left gland, arrow); note the nuclear RARα location. RXRα was also detected in almost all PIN cells (Fig. 1H, arrow). An attempt was made to compare the percentage of RARα and RXRα in placebo- and 9cRA-treated animals, but no difference was found (data not presented), suggesting a receptor independent mechanism of cell growth inhibition. To further understand whether 9cRA suppresses prostate carcinogenesis by inhibiting cell proliferation or/and by inducing apoptosis and/or cellular senescence (CS), mice treated with MNU and MNU + 9cRA (Table 3) were sacrificed at the end of the experiment, DLP and AP were frozen on dry ice, and then frozen tissue sections were prepared and treated with SA-β-Gal kit for identification of senescent cells. Cell proliferation was determined on frozen sections by Ki-67 antibody (Fig. 2A and 2B). The values of Ki-67 positive cells not associated with PIN in p27+/- and p27-/- control (placebo-treated) mice are close, with a lack of statistical difference between them: 1.4 ± 0.3% vs. 1.7 ± 0.8% (p > 0.5), respectively (Table 4). In PIN of p27-/- mice, Ki-67 values are much higher as compared to those of p27+/- mice: 4.6 ± 1.4% vs. 2.6 ± 0.4% (p < 0.005), respectively. 9cRA was not efficacious and did not affect proliferating cells in glandular structures not associated with PIN (Fig. 2B). However, 9cRA decreased Ki-67 positive cells in PIN of both p27+/- and p27-/- mice from 2.6 ± 0.4% (placebo) to 1.5 ± 0.5% (p < 0.01) and from 4.6 ± 0.4% (placebo) to 3.5 ± 0.5% (p < 0.001), respectively. Apoptosis was rare in both PIN and non-PIN epithelial cells of placebo-treated animals and 9cRA did not affect their number (Table 5). Surprisingly, a high percentage of senescent cells was found in PIN and non-PIN prostate epithelial cells of both placebo- and 9cRA-treated mice (Fig. 2E-H). 9cRA induced CS in non-PIN epithelial cells of both p27+/- and p27-/- mice: from 0.8 ± 0.4% in placebo to 2.7 ± 1.3% in 9cRA treated for p27+/- mice and from 1.2 ± 0.4% in placebo to 2.4 ± 0.5% in 9cRA-treated p27-/- mice. 9cRA also induced CS in PIN of both p27+/- and p27-/- mice: from 1.0 ± 0.5% in placebo to 4.8 ± 1.2% in 9cRA-treated p27+/- mice (p < 0.001) and from 3.2 ± 0.7% in placebo to 6.8 ± 2.2% (p < 0.001) in 9cRA-treated p27-/- mice (Fig. 2G-H).

**Table 5:** 9cRA inhibits cell proliferation (Ki-67) and induces senescent phenotype in PIN

Genotype	Animals	Cell Type	9cRA	Ki-67%	p	Apo-%	p	Sen-%	p
p27+/+	5	PEC	0	0.8 ± 0.3*		0.4 ± 0.2		0.8 ± 0.4	
p27+/+	6	PEC	+	0.6 ± 0.4	ns	0.6 ± 0.2		1.1 ± 0.5	
p27+/+	5	PIN	0	1.2		0.3		0.9	
p27+/+	5	PIN	+	NA		NA		NA	
p27+/-	6	PEC	0	1.4 ± 0.4* **	0.01	0.3 ± 0.2		0.8 ± 0.4	
p27+/-	6	PEC	+	1.2 ± 0.5	ns	0.4 ± 0.2	ns	2.7* ± *1.3	0.001
p27+/-	5	PIN	0	2.6 ± 0.4		0.6 ± 0.3		1.0 ± 0.5	
p27+/-	6	PIN	+	1.5 ± 0.5	0.001	0.5 ± 0.4	ns	4.8 ± 1.2	0.001
p27-/-	6	PEC	0	1.7 ± 0.5* **		0.3 ± 0.2		1.2 ± 0.4	
p27-/-	5	PEC	+	1.6+0.7	ns	0.4 ± 0.2	ns	2.5* ± *1.3	0.001
p27-/-	6	PIN	0	4.6 ± 0.4		0.6 ± 0.3		3.2 ± 0.7	
p27-/-	5	PIN	+	3.5 ± 0.5	0.001	0.5 ± 0.4	ns	6.8 ± 2.2	0.001

The above data were obtained from placebo- and 9cRA-treated animals at the time of their sacrifice at 9-12 months of age. PEC, prostate epithelial cells from DLP not associated with PIN; 0, Placebo-treated animals; +, animals treated with 9cRA; PIN (low and high grade) was identified in H&E stained slides. To determine the incidence (%) and frequency of PIN in DLP and AP, 4 μm thick paraffin sections were cut longitudinally through the urethral part of the prostate stepwise at three separate levels 30 μm apart. All resulting slides were labeled consecutively. Proliferating cells were detected by Ki-67 antibody (Ki-67); Cells in apoptosis (Apo-%) were detected by ApopTag kit based on the TUNEL assay; Cells in senescence (Sen-%) were identified by SA-β-Gal staining employing a kit from Cell Signaling. At least 500 PEC or PIN cells were evaluated to determine the percentage of proliferating (Ki-67-%), apoptotic (Apo-%) or cells in senescence (Sen-%); * or ** indicates significant difference (p < 0.05) in the values between corresponding groups.

## Discussion

The main goal of this study was to determine whether p27 deficiency in p27+/- and p27-/- mice may affect PEC proliferation and carcinogenesis, as well as the efficacy of 9cRA in suppressing the neoplastic process. p27 heterozygous (+/-) and p27 null (-/-) mice were selected for the following reasons: a) the difference in p27^Kip1 ^expression between p27+/+, p27+/- and p27-/- mice simulates the gene's alterations in the course of prostate cancer development in humans, as p27^Kip1 ^is expressed in normal PEC, lacking in BPH, and differentially expressed in malignant tumors [4,8]; b) p27-/- mice develop predominantly hyperplastic and premalignant (PIN) lesions, which are much easier to suppress by cancer preventive agents, like retinoids, than aggressively growing carcinomas such as those in PTEN mice [15-17]; c) previous studies have shown that p27 haplo-insufficiency promotes carcinogen- and radiation-induced tumors in various organs [19,20]; d) retinoids are efficacious inhibitors of prostate carcinogenesis in animal models [23,24]; and e) p27^Kip1 ^is involved in mediating the effect of retinoids on PEC both *in vitro *and *in vivo *[25-27]. Here, we confirmed two of our previous observations [28,31] and those of others [32-35]. First, we confirmed that p27-/-mice are larger than p27+/+ mice, whereas heterozygous p27+/- mice have an intermediate body weight (see Table 2). The increase in body weight in p27-/- mice was due to generalized organomegaly with disproportional enlargement of the spleen, thymus, testis, ovaries, pituitary gland (data not shown). Second, we confirmed that old (> 7-12 months) p27-/- and p27+/- mice develop spontaneous hyperplasia and adenomas in the intermediate pituitary lobe, intestine, kidney and thymus that lead to the animals' death. The data generated in this study indicates that under normal physiological conditions, PEC proliferation in two-month-old mice is similar in all three p27 genotypes (Table 1). However, when the values of proliferating cells were compared in 9- to12-month-old mice, proliferating cells were much higher in p27-/- and p27+/- than in p27+/+ mice, suggesting that p27 absence or insufficiency promotes PEC proliferation in the aging animals where hyperplastic and pre-malignant lesions occur (Table 5). This data supports previous studies from Cardon-Cardo's group [7], which found higher PEC proliferation (Ki-67 labeling) in p27 null (-/-) mice as compared to p27+/+ mice. However, they did not provide information whether p27 heterozygous and null mice develop PIN and whether the frequency of PIN could be regulated by hormone stimulation or cancer prevention agents. Thus, it appears that the loss of p27^Kip1 ^keeps a high base level of PEC proliferation in old animals that leads to development of hyperplastic, pre-malignant and malignant lesions. When PEC proliferation was stimulated by testosterone, a much higher proliferative response was observed in p27+/- and p27-/- than in p27+/+ mice, indicating that p27^Kip1 ^lack or haplo-insufficiency increases the sensitivity of PEC to hormone stimulation. This information is important, because there are sufficient clinical data showing that an increased testosterone circulation level in men is associated with increased risk in developing prostate cancer [16]. Therefore, temporary or continuous down-regulation of p27^Kip1 ^expression in PEC may promote the effect of testosterone or other growth factors in stimulating prostate carcinogenesis.

In a previous study Fero et al. [19] reported that p27^Kip1 ^protein expression was lacking in p27-/- mice, decreased by 50% in p27+/- mice, and promoted radiation and chemical-induced (N-Ethyl-N-Nitrosourea-ENU) carcinogenesis. In p27-/- mice, tumors developed in the lung, intestine, pituitary, ovary, and endometrium earlier and at a higher frequency as compared to both p27+/- and p27+/+ mice. However, in Fero's study prostate and male reproductive organs were not examined at the tissue level (personal communication) and neither PEC proliferation was stimulated by testosterone. In another study, Di Cristofano et al. [15] reported on cooperation between p27^Kip1 ^and PTEN in developing PIN and prostate cancer. PTEN is a tumor suppressor and its activity leads to the induction of p27^Kip1^; therefore, the lack or down-regulation of PTEN may suppress p27^Kip1 ^expression and thus promote cell proliferation and carcinogenesis. Concomitant inactivation of one PTEN allele and one or both p27 alleles in transgenic mice accelerates the development and progression of prostate and other malignancies [16,17]. We did not use PTEN+/-, p27-/- mice in this study because tumors in the prostate and other organs develop very early, within 3 months after birth, and most animals die at the age of 16-20 weeks. This very aggressive neoplastic process is difficult to modulate with retinoids and other cancer prevention agents. We found that the progressive decrease of p27^Kip1 ^expression in p27+/- and further in p27-/- mice promotes MNU-induced prostate carcinogenesis (Table 3). It appears that there is a negative correlation between the level of p27^Kip1 ^expression and the incidence and multiplicity of PIN and prostate tumors. Since most animals were sacrificed between 9 and 12 months of age, this time period is probably not sufficient for PEC in p27+/+ mice to transform in PIN and progress in invasive carcinomas. For instance, studies on rats treated with MNU have shown that prostate pre-malignant lesions and carcinomas occur 12 to 18 months after carcinogen administration [23,24]. To accelerate prostate carcinogenesis, we treated p27+/+, p27+/- and p27-/- mice with MNU and stimulated PEC proliferation by testosterone + estradiol releasing pellets (Table 4). Previous studies, including ours, have shown that testosterone + estradiol stimulate PEC proliferation [35] and carcinogenesis in rats [23,24]. However, because of the severe prostate hypertrophy with complete urethra blockage in some animals and high death rate, the study continued with surviving animals only. Hormone stimulation was more efficacious in inducing PIN and tumors in p27+/- and p27-/- mice than in p27+/+ mice, suggesting that deregulation of p27^Kip1 ^expression in PIN may promote hormone mediated prostate carcinogenesis in men. The data for multiplicity of PIN in Table 3, where MNU was only used to induce prostate carcinogenesis, are close to those in Table 4, where MNU + testosterone + estradiol were employed. This can be explained by termination of hormone stimulation which occurs 60 days after pellet implantation and which may cause cell death and thereby eliminate transformed PEC and their potential progression towards PIN and tumors.

What is most interesting from this study was that 9cRA suppressed MNU-induced prostate carcinogenesis but was unable to suppress MNU+hormone-stimulated carcinogenesis. Even in p27+/- mice, a tendency for an increase of PIN by 9cRA is obvious, although the differences in values with placebo-treated animals were not significant. In all three p27 genotypes, the values of PIN incidence are close in placebo- and 9cRA-treated animals. This data supports the results from clinical trials where patients with an increased risk of prostate cancer have been treated with beta carotene, retinol or other antioxidants [36,37]. No efficacy on prostate cancer reduction has been found, but an increase in lung cancer incidence has been observed that led to preliminary termination of a clinical trial. One potential hypothesis is that 9cRA or its metabolites are not efficacious in highly proliferating cell systems, like PEC stimulated by the above hormones. Our data from short-term experiments where mice have been treated with testosterone + 9cRA supports this hypothesis. Another alternative is that in long-term experiments 9cRA may protect PEC from cell death after expiration of testosterone + estradiol stimulation (60-day releasing pellets). However, in the long-term carcinogenesis study, 9cRA suppressed the incidence and frequency of PIN in MNU-treated mice, indicating that a lack of or decreased p27^Kip1 ^level does not affect the efficacy of 9cRA in suppressing prostate carcinogenesis. 9cRA is a ligand of both RARs α,β,γ and RXRs α,β,γ, suggesting that it may affect various cellular functions by receptor dependent mechanisms [22,25,38]. By ICH we found that RARα and RXRα, principal targets of retinoids and rexinoids, respectively, are expressed in PEC of all three p27 genotypes, suggesting a potential receptor-dependent mechanism of 9cRA-induced inhibition of PEC proliferation and carcinogenesis. An attempt was made to compare RARα and RXRα positive cells in control- and 9cRA-treated animals in a carcinogenesis study (Table 3). However, no difference in the percentage of receptor positive cells was found (data not presented), suggesting receptor independent mechanisms of 9cRA-induced inhibition of prostate carcinogenesis [39]. It has been shown that retinoids, including 9cRA, at pharmacological doses preferentially suppress cell proliferation and may also induce cell differentiation [40]. Some previous studies have also shown that retinoids may induce apoptosis as well, depending on the tissue type and the dose used [9,15]. Our data obtained from the carcinogenesis study indicates that 9cRA suppresses cell proliferation but is unable to induce apoptosis. Interestingly, 9cRA did not significantly affect cell proliferation in non-PIN cells, which have low proliferative activity, but significantly reduced proliferating cells in PIN of both p27+/- and p27-/- mice, indicating that 9cRA may have the potential to suppress preferentially pre-malignant stages of prostate carcinogenesis in men independent of the levels of p27^Kip1 ^expression. Surprisingly, we found that in addition to inhibition of cell proliferation, 9cRA can also induce CS in both PIN and non-PIN associated PEC (Table 5 and Fig. 2E-H). In a previous study on mammary carcinogenesis, we compared the effect of 9cRA and 4-HPR on CS in MNU-induced mammary tumors and found that 9cRA has a superior role [24]. Data from human prostate also suggest that senescent cells are frequently detected in BPH and rarely in prostate cancer, suggesting that malignant transformation of prostate epithelial cells is associated with loss of the potential to senesce [8,41].

## Conclusion

Our data indicate that p27^Kip1 ^deficiency in PEC promotes cell proliferation in an age-dependent manner and increases cellular response to hormone stimulation. p27^Kip1 ^haplo-insufficiency and deficiency stimulate MNU-induced prostate carcinogenesis, suggesting that PIN in human prostate lacking partial or total p27^Kip1 ^expression may have a higher potential to progress and develop malignant phenotype than lesions with wild type p27. The efficacy of 9cRA in suppressing PIN is apparently not p27-dependent, indicating that in potential clinical trials, 9cRA may affect pre-malignant and tumor cells that differentially expressed p27^Kip1^. The induction of CS by 9cRA in PIN suggests that this biomarker could be used as a potential biomarker of response in clinical trials for the prevention and treatment of prostate cancer.

## Competing interests

The authors declare that they have no competing interests.

## Authors' contributions

WT ran animal experiments and performed p27 gene characterization. AM was involved in animal experiments, p27 gene analysis and hormone stimulation of prostate cells. AA was involved in identification of p27 in prostate cells. HK was involved in the initiating phase of this study and conducted animal studies and p27 gene analysis. NS performed immunocytochemistry and evaluated proliferating cells. AS performed most of the immunocytochemistry for identification of cell proliferation, apoptosis, and cellular senescence. AG was involved in animal experiments and development of p27+/+, p27+/- and p27-/- mice. HK provided p27+/- and p27-/- mice and helped with identification of their genotype. KC was PI of the study and planned, organized, and supervised animal experiments, hormone stimulation of prostate, the carcinogenesis study, and evaluation of morphological alterations in various prostate lobes; KC also evaluated the data and wrote the manuscript, which all authors have read and approved.

## Pre-publication history

The pre-publication history for this paper can be accessed here:

http://www.biomedcentral.com/1471-2407/10/541/prepub
